# Focused ultrasound-induced blood-brain barrier opening improves adult hippocampal neurogenesis and cognitive function in a cholinergic degeneration dementia rat model

**DOI:** 10.1186/s13195-019-0569-x

**Published:** 2019-12-27

**Authors:** Jaewoo Shin, Chanho Kong, Jihyeon Lee, Bo Young Choi, Jiyeon Sim, Chin Su Koh, Minkyung Park, Young Cheol Na, Sang Won Suh, Won Seok Chang, Jin Woo Chang

**Affiliations:** 1 Department of Neurosurgery, Yonsei University College of Medicine, Seoul, 03722 Republic of Korea; 2 Brain Korea 21 PLUS Project for Medical Science and Brain Research Institute, Yonsei University College of Medicine, Seoul, 03722 Republic of Korea; 3 Department of Physiology, Hallym University College of Medicine, Chuncheon, 24252 Republic of Korea; 4 Department of Neurosurgery, Catholic Kwandong University College of Medicine, International St Mary’s Hospital, Incheon Metropolitan City, 22771 Republic of Korea

**Keywords:** Rats, Sprague-Dawley, Brain-derived neurotrophic factor, Microbubbles, Sonication, Hippocampus, Dementia, Alzheimer’s disease, Neuropathology

## Abstract

**Background:**

The persistence of adult hippocampal neurogenesis (AHN) is sharply decreased in Alzheimer’s disease (AD). The neuropathologies of AD include the presence of amyloid-β deposition in plaques, tau hyperphosphorylation in neurofibrillary tangles, and cholinergic system degeneration. The focused ultrasound (FUS)-mediated blood-brain barrier opening modulates tau hyperphosphorylation, the accumulation of amyloid-β proteins, and increases in AHN. However, it remains unclear whether FUS can modulate AHN in cholinergic-deficient conditions. In this study, we investigated the effect of FUS on AHN in a cholinergic degeneration rat model of dementia.

**Methods:**

Adult male Sprague-Dawley rats (*n* = 48; 200–250 g) were divided into control (phosphate-buffered saline injection), 192 IgG-saporin (SAP), and SAP+FUS groups; in the two latter groups, SAP was injected bilaterally into the lateral ventricle. We applied FUS to the bilateral hippocampus with microbubbles. Immunohistochemistry, enzyme-linked immunosorbent assay, immunoblotting, 5-bromo-2′-deoxyuridine labeling, an acetylcholinesterase assay, and the Morris water maze test were performed to assess choline acetyltransferase, acetylcholinesterase activity, brain-derived neurotrophic factor expression, neural proliferation, and spatial memory, respectively. Statistical significance of differences in between groups was calculated using one-way and two-way analyses of variance followed by Tukey’s multiple comparison test to determine the individual and interactive effects of FUS on immunochemistry and behavioral analysis. *P* < 0.05 was considered significant.

**Results:**

Cholinergic degeneration in rats significantly decreased the number of choline acetyltransferase neurons (*P* < 0.05) in the basal forebrain, as well as AHN and spatial memory function. Rats that underwent FUS-mediated brain-blood barrier opening exhibited significant increases in brain-derived neurotrophic factor (BDNF; *P* < 0.05), early growth response protein 1 (EGR1) (*P* < 0.01), AHN (*P* < 0.01), and acetylcholinesterase activity in the frontal cortex (*P* < 0.05) and hippocampus (*P* < 0.01) and crossing over (*P* < 0.01) the platform in the Morris water maze relative to the SAP group after sonication.

**Conclusions:**

FUS treatment increased AHN and improved spatial memory. This improvement was mediated by increased hippocampal BDNF and EGR1. FUS treatment may also restore AHN and protect against neurodegeneration, providing a potentially powerful therapeutic strategy for AD.

## Background

Adult neurogenesis appears to be restricted to two regions, i.e., the subventricular zone (SVZ) of the lateral ventricles and the subgranular zone (SGZ) of the hippocampal dentate gyrus (DG). Importantly, adult hippocampal neurogenesis (AHN) was first reported over 50 years ago by Altman and Das [1], and newborn neurons are generated continuously throughout life in the mammalian brain, including the human brain [2, 3]. Since then, numerous studies have reported that AHN is implicated in cognition and endogenous repair mechanisms in normal physiological conditions such as learning and memory [4]. Interestingly, according to the recent research, the persistence of AHN appears to be decreased in aged adults and Alzheimer’s disease (AD) [5, 6].

AD is one of the major causes of age-related dementia and is characterized by cognitive impairment, amyloid-β deposition in plaques, tau hyperphosphorylation in neurofibrillary tangles, loss of synapses, loss of neuronal cells, and cholinergic dysfunction [7]. Dysfunction of the basal forebrain cholinergic (BFC) system, a significant characteristic of AD, induces neuropathological changes before clinical symptoms manifest [8–10]. The hippocampus and cortex receive gamma-aminobutyric acidergic, glutamatergic, and cholinergic input from the basal forebrain of the medial septum-diagonal band complex (MS/DB) [11, 12]. Thus, lesions in, or the inactivation of, cholinergic neurons in MS/DB result in a decrease of acetylcholinesterase (AChE) and choline acetyltransferase (ChAT), consequently diminishing AHN [13–16].

Despite intensive research efforts, none of the currently available treatments for AD can completely cure or prevent the course of age-related cognitive impairment, and the pathological mechanism is not clearly understood. Numerous pharmacological therapies have been developed to treat AD [17]. However, 98% of small-molecule drugs (< 400 Da) and 100% of large-molecule drugs (> 500 Da) cannot cross the blood-brain barrier (BBB) [18], making the prevention and treatment of brain disorders difficult.

Focused ultrasound (FUS) combined with contrast agent microbubbles is a noninvasive technique that transiently opens BBB in targeted regions, thereby enabling localized therapeutic drug, gene, or nanoparticle delivery into the brain for treating central nervous system (CNS) disorders [19–21]. Considering that drugs that have been, or are currently being, developed for AD are mostly large molecules, FUS may enhance the effects of these drugs especially in patients with early-stage AD who have an intact BBB [22]. Moreover, several reports suggest that FUS stimulates neuronal activity and modulates proteomes and transcriptomes, independent of any therapeutic agent [23–25].

Previous studies indicate that FUS-mediated BBB opening can modulate the accumulation of amyloid-β and tau hyperphosphorylation in AD transgenic mice and increase AHN in wild-type mice [26–30]. Recently, Moreno-Jiménez et al. reported the persistence of AHN in human DG of subjects aged over 90 years; however, the number and maturation of immature neurons in DG sharply decreased in patients with AD. This finding has gained attention for potential therapeutic strategies as an underlying memory impairment in AD [31]. However, it remains unclear whether FUS can modulate AHN in a cholinergic-deficient condition. In this study, we investigated the effect of FUS on AHN and the cholinergic system in a cholinergic degeneration dementia rat model, which is a key pathogenic feature of dementia. Furthermore, if FUS was effective in increasing AHN, the synergistic effects of AHN modulation and drug delivery could improve treatment outcomes of AD.

## Materials and methods

### Ethical considerations

All animal experimental procedures were conducted in compliance with the Guide for the Care and Use of Laboratory Animals of the National Institutes of Health and were approved by the Institutional Animal Care and Use Committee (IACUC; 2016-0339) of Yonsei University. Animals were housed in groups of three in laboratory cages with food and water available ad libitum in a 12-h light/dark (lights on at 07:00) cycle in a room with controlled temperature (22 ± 2 °C) and humidity (55 ± 5%).

### Rat model generation

**Fig. 1:**
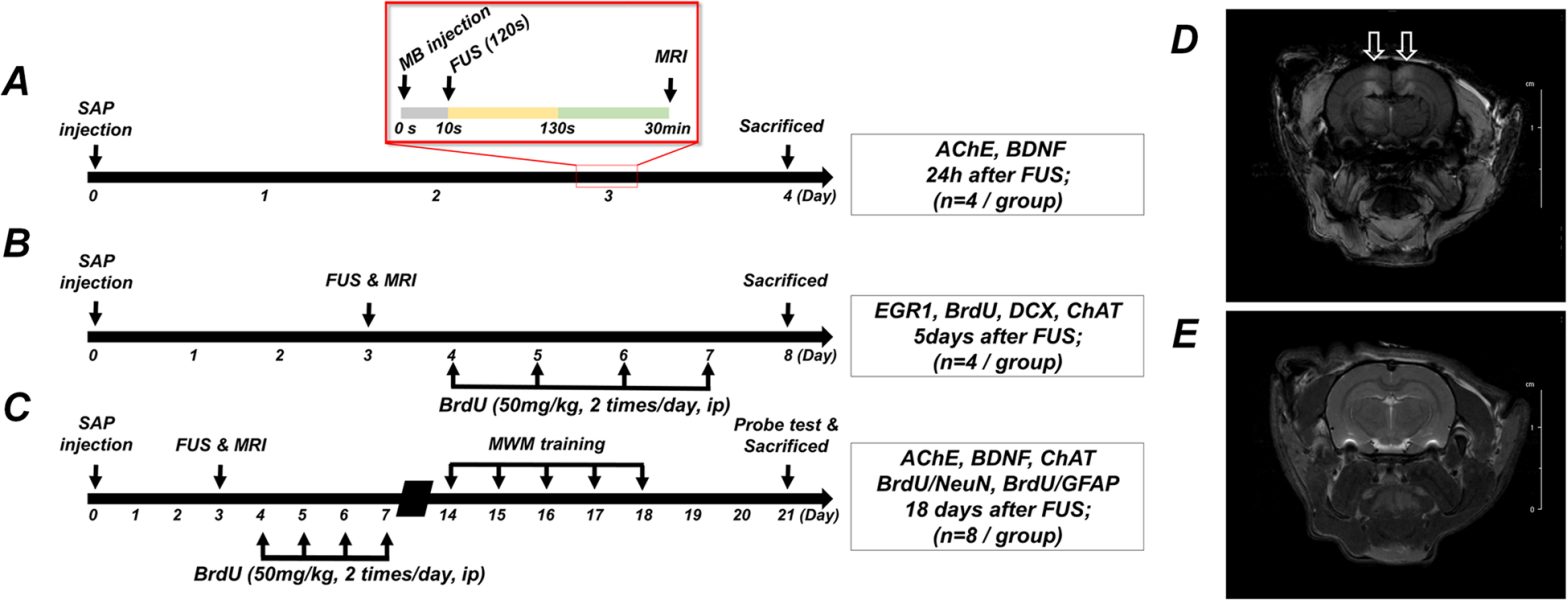
Schematic of the experimental procedure and FUS system. **a** Timeline of the focused ultrasound (FUS) experiment for the analysis of AChE activity and BDNF expression 24 h after sonication (control, *n* = 4; 192 IgG-saporin [SAP], *n* = 4; SAP+FUS, *n* = 4). **b** BrdU and doublecortin (DCX) 5 days after sonication (control, *n* = 4; SAP, *n* = 4; SAP+FUS, *n* = 4). **c** AChE, BDNF, BrdU/NeuN, and BrdU/GFAP 18 days after sonication (control, *n* = 8; SAP, *n* = 8; SAP+FUS, *n* = 8). **d** Confirmed FUS-mediated blood-brain barrier (BBB) opening with MRI. Gadolinium-enhanced T1-weighted images show contrast enhancement. Arrow indicates regions of BBB opening. **e** Confirmed FUS-mediated edema with T2-weighted MRI

Previous studies have modeled cholinergic degeneration and cognitive function-impaired dementia in rats by intraventricularly administering the selective immunotoxin 192 IgG-saporin (SAP) to induce lesions in BFC neurons [32–35]. To investigate the effect of FUS on AHN in a cholinergic degeneration rat model of dementia, adult male Sprague Dawley rats (*n* = 48; 200–250 g) were divided into control (phosphate-buffered saline [PBS] injection), SAP, and SAP+FUS groups. The dementia rat model (SAP, *n* = 16; SAP+FUS, *n* = 16) was generated by injecting SAP (Chemicon, Temecula, CA, USA), and the control group (*n* = 16) received a bilateral ventricular infusion of 1× PBS (PH 7.4) into the brain. All 48 rats were anesthetized with a mixture of ketamine (75 mg/kg), xylazine (Rompun™; 4 mg/kg), and acepromazine (0.75 mg/kg) and were fixed in a stereotaxic frame. As previously described, scalp skin was incised, and two holes were drilled into the skull at the following coordinates: from the bregma anterior-posterior, − 0.8 mm; medial-lateral, ± 1.2 mm; and dorsal-ventral, − 3.4 mm [36]. Thereafter, 4 μl of SAP (0.63 μg/μl) was bilaterally injected at a rate of 1 μl/min into the lateral ventricle of the rats in the SAP and SAP+FUS groups using a syringe pump (Legato 130, 788130, KD Scientific, Holliston, MA, USA). As shown in Fig. 1a, rats were sacrificed at different time points, i.e., 24 h, 5 days, and 18 days after FUS. To detect changes in AChE and BDNF expression levels, observe proliferation and neuroblast production, and observe neuronal differentiation of BrdU-positive cells and long-term effects of AChE and BDNF, the rats were sacrificed 24 h, 15 days, and 18 days after FUS, respectively.

### Focused ultrasound

The pulsed ultrasound was generated using a 0.5-MHz single-element spherically focused transducer (H-107MR, Sonic Concept Inc., Bothell, WA, USA) with a diameter of 51.7 mm and radius of curvature of 63.2 mm. A waveform generator (33220A, Agilent, Palo Alto, CA, USA) was connected to a 50-dB Radio Frequency Power Amplifier (240 L, ENI Inc., Rochester, NY, USA) to drive the FUS transducer, and a power meter (E4419B, Agilent) was used to measure the input electrical power. The transducer electrical impedance was matched to the output impedance of the amplifier (50 Ω) with an external matching network (Sonic Concept Inc., Bothell, WA, USA). A cone filled with distilled, degassed water was mounted onto the transducer assembly (Additional file 1: Figure S1). A needle-type hydrophone (HNA-0400, Onda, Sunnyvale, CA, USA) was used for the transducer calibration, which measured the acoustic beam profile in the tank filled with degassed water. The transducer was mounted on the cone filled with degassed water, and the end of its tip was wrapped in a polyurethane membrane.

The experimental procedure is shown in Fig. 1. Briefly, rats were anesthetized with a mixture of ketamine (75 mg/kg) and xylazine (4 mg/kg), and their heads mounted on a stereotaxic frame (Narishige, Tokyo, Japan) with ear and nose bars. Ultrasound transmission gel (ProGel-Dayo Medical Co., Seoul, South Korea) was used to cover the area between the animal’s skull and the cone tip to maximize the transmission efficiency of the ultrasound. FUS was targeted bilaterally to the region containing the hippocampus according to the 3D positioning system. DEFINITY® microbubble contrast agents (mean diameter range, 1.1–3.3 μm; Lantheus Medical Imaging, North Billerica, MA, USA) were diluted in saline and injected intravenously into the tail vein 10 s before sonication. Sonication parameters were set as follows: burst duration, 10 ms; pulse repetition frequency, 1 Hz; total duration, 120 s; and average peak-negative pressure, 0.25 MPa.

### Magnetic resonance imaging

After sonication, magnetic resonance imaging (MRI) experiments were performed with a Bruker 9.4 T 20-cm-bore MRI system (Biospec 94/20 USR; Bruker, Ettlingen, Germany) and a rat head coil. A gadolinium-based contrast agent, gadobutrol (Gd, Gadovist; Bayer Schering Pharma AG, Berlin, Germany; 0.2 mL/kg), was injected into the tail vein, and contrast-enhanced T1-weighted images were used to confirm the BBB opening from the FUS. T1-weighted MRI was performed with and without the use of gadobutrol contrast (Fig. 1d). T2-weighted images were used to confirm edema with FUS (Fig. 1e). Sequence parameters are summarized in Table 1.

**Table 1:** Sequences and parameters of MRI

	T1-weighted Imaging	T2-weighted Imaging
Echo	1	1
TR (ms)	350	2500
TE (ms)	5.4	33
FA (deg)	40	180
NEX	2	2
FOV (cm)	3.5	3.5
Matrix	256 × 256	256 × 256

*TR* repetition time, *TE* time to echo, *FA* fractional anisotropy, *NEX* number of excitations, *FOV* field of view

### Behavioral test—Morris water maze

Rats underwent the Morris water maze (MWM) test at 2 weeks after receiving SAP injection. The MWM apparatus comprised a circular pool (diameter, 2 m; height, 50 cm) filled to a depth of 30 cm with dark water (23 °C). A concealed black, round platform (diameter, 15 cm) was situated 1–2 cm below the surface of the water in the center of a target quadrant. All rats were trained for four trials per day for 5 consecutive days. During training, the location of the hidden platform was fixed, and spatial cues were provided for guidance. For each training trial, the rats were placed in the water facing the wall at one of the four starting points and were given 60 s to reach the hidden platform. After finding the platform, the rats were allowed to remain on the platform for 10 s. The rats that could not find the platform within 60 s were led to the platform by the experimenter and were allowed to remain on the platform for 10 s. The rats were given a 60-s probe test without the platform 72 h after the last training trial. Swimming speed, swim path, time spent in each zone, and distance swam were recorded using the SMART video-tracking system (Harvard Apparatus, Holliston, MA, USA).

### BrdU labeling

To investigate the effect of FUS on neurogenesis, animals were injected intraperitoneally with 5-bromo-2′-deoxyuridine (BrdU; Sigma-Aldrich, St. Louis, MO, USA), used for the detection of proliferating cells, twice a day for 4 consecutive days, 24 h after sonication [30, 37].

### Histological evaluation

#### Brain tissue preparation

The animals were sacrificed 5 days (*n* = 4 per group) or 18 days (*n* = 4 per group) after FUS sonication. The rats were anesthetized via the intraperitoneal injection of a mixture of ketamine (75 mg/kg) and xylazine (4 mg/kg). For the blood wash-out and brain fixation, transcranial perfusion was performed with 0.9% normal saline and 4% paraformaldehyde in 1× PBS. After perfusion, all brains were post-fixed in 4% paraformaldehyde for 1 h. Subsequently, the brain tissue was transferred to a 30% sucrose solution for 3 days. The brains were then sectioned into 30-μm-thick slices using a Leica CM1850 cryostat (Leica Biosystems, Wetzlar, Germany).

#### Immunohistochemistry

To determine the effects of FUS on cell proliferation, 24 h after sonication, brain sections were incubated in 0.3% H_2_O_2_ for 15 min to inactivate endogenous peroxidase activity. DNA denaturation was then performed by antigen retrieval in 2N HCl at 37 °C for 90 min and neutralization twice with 0.1 M borate buffer for 10 min. The sections were then washed with PBS, blocked with 5% normal goat serum for 1 h, and incubated overnight at 4 °C with the following monoclonal antibodies diluted in PBS containing 0.3% normal goat serum and 0.3% Triton X-100: mouse anti-BrdU (1:150, BMC9313, Roche Molecular Biochemicals, Mannheim, Germany), rabbit anti-early growth response 1 antibody (EGR1; 1:200, 4153S, Cell Signaling Technology, Inc., Beverly, MA, USA), goat anti-DCX (1:200, SC8066, Santa Cruz Biotechnology, Inc., Santa Cruz, CA, USA), and goat anti-ChAT (1:100, AB144P, Millipore, Bedford, MA, USA). Thereafter, the sections were incubated with affinity-purified biotinylated goat anti-mouse IgG secondary antibodies (1:400, BA-9200, Vector Laboratories, Burlingame, CA, USA), affinity-purified biotinylated rabbit anti-goat IgG secondary antibodies (1:400, BA-5000, Vector Laboratories, CA, USA), affinity-purified biotinylated goat rabbit anti-IgG secondary antibodies (1:400, BA-1000, Vector Laboratories, CA, USA), and affinity-purified biotinylated according to the avidin-biotin complex method (ABC Elite; Vector Laboratories, CA, USA). Immunoreactivity was evaluated using a DAB substrate kit (Thermo Fisher Scientific, Fremont, CA, USA). EGR1 was counterstained using hematoxylin (H-3401, Vector Laboratories, CA, USA). The samples were examined using an optic microscope (BX51; Olympus, Tokyo, Japan).

#### Immunofluorescence staining

The sections were double stained with BrdU and NeuN or BrdU and GFAP and then incubated for 2 h in a mixture of mouse monoclonal anti-BrdU (1:150, BMC9313, Roche Molecular Biochemicals, Mannheim, Germany) and either rabbit polyclonal anti-NeuN (Neuronal nuclei; 1:500, ABN78, Millipore, Bedford, MA, USA) or goat polyclonal anti-GFAP (Glial fibrillary acidic protein; AB7260 1:200, Abcam Cambridge, MA, USA). This was followed by a 2-h incubation in a mixture of goat anti-mouse Alexa Fluor® 594 IgG (1:500, A11005, Invitrogen, Carlsbad, CA, USA) (BrdU) and goat anti-rabbit Alexa Fluor® 488(1:500, A11008, Invitrogen, Carlsbad, CA, USA) (NeuN, GFAP) at room temperature. Fluorescence signals were confirmed using a Zeiss LSM 710 confocal imaging system (Carl Zeiss, Oberkochen, Germany) with a sequential scanning mode for Alexa 594 and 488. Stacks of images (1024 × 1024 pixels) from consecutive 0.9–1.2-μm-thick slices were obtained by averaging eight scans per slice. The resulting images were processed with ZEN 2010 (Carl Zeiss).

#### Quantification of cell counting

Seven coronal sections (185-μm intervals) from each animal, collected from 3.2 to 4.5 mm posterior to the bregma, were analyzed to quantify the BrdU-, DCX-, and EGR1-positive cells. The sections were photographed using a virtual microscope (BX51; Olympus) with a × 10 objective. Coded sections were counted by a blinded observer who quantified the number of BrdU- and DCX-positive cells in the bilateral subgranular zone (SGZ) and granular cell layer (GCL) of the dentate gyrus (DG) and the number of EGR1-positive cells in the bilateral CA1, CA3, and DG of the hippocampus. To analyze the phenotype of BrdU-positive cells, we determined whether BrdU-positive cells in the SGZ and GCL (SGZ/GCL) expressed NeuN or GFAP with confocal microscopy. A double-positive percentage was calculated as BrdU+/NeuN+ or BrdU+/GFAP+ for total BrdU-positive cells in the SGZ/GCL.

### ELISA and immunoblotting

#### Brain sample preparation

At 24 h (*n* = 12) and 18 days (*n* = 12) after sonication, the remaining rats from each group were anesthetized with a mixture of ketamine (75 mg/kg), xylazine (4 mg/kg), and acepromazine (0.75 mg/kg). They were then decapitated with a guillotine, and their brains removed. The prefrontal cortex and hippocampus regions were dissected with fine forceps to yield 1-mm coronal brain slices using a rat brain slicer matrix. These samples were homogenized in a Kontes glass homogenizer (Kontes Glass Co., Vineland, NJ, USA) with a protein extraction solution containing 1.0 mM PMSF, 1.0 mM EDTA, 1 μM pepstatin, 1 μM leupeptin, and 1 μM aprotinin (PRO-PREP, Catalog no. 17081, iNtRON Biotechnology, Seongnam, Korea). After extraction, the slices were centrifuged for 20 min at 12,000 rpm. The total protein concentration was measured using the bicinchoninic acid protein assay reagent kit (Pierce, Rockford, IL, USA). All extraction steps were performed at 4 °C, and protein samples were stored at − 80 °C until use.

#### AChE assay

To evaluate the enzymatic activity of AChE, the modified version of the method of Ellman et al. was used [38]. In brief, 20-μl triplicate samples were mixed with a reaction mixture (0.2 mM 5, 5′-dithiobis (2-nitrobenzoic acid) [Sigma-Aldrich], 0.56 mM acetylthiocholine iodide [Sigma-Aldrich], 10 μM tetraisopropyl pyrophosphoramide [Sigma-Aldrich], and 39 mM phosphate buffer; pH 7.2) at 37 °C for 30 min. The quantification of optical density was performed at a wavelength of 405 nm.

#### Western blot analysis

Western blot analyses were performed on the same protein samples as those used for the AChE assay. Twenty micrograms of each protein were separated by 12% sodium-dodecyl-sulfate-polyacrylamide gels and electrotransferred onto polyvinylidene fluoride membranes using a Bio-Rad miniature transfer apparatus for 100 min at 0.3 A. The membranes were then blocked using a blocking buffer (5% non-fat dry milk in PBS containing 0.05% Tween 20) for 1 h at room temperature (25 °C). The membranes were then incubated with primary antibodies overnight at 4 °C with rabbit monoclonal anti-brain-derived neurotrophic factor (BDNF, 1:1000; Abcam, Cambridge, UK) and mouse monoclonal anti-ß-actin (1:10000; Sigma-Aldrich). The corresponding secondary antibodies were then applied for 90 min at room temperature with goat anti-rabbit IgG(H+L)-HRP (1:2000 at BDNF; GenDEPOT, Katy, TX, USA) and goat anti-mouse IgG(H+L)-HRP (1:10000; GenDEPOT). The proteins were visualized using an enhanced chemiluminescence solution (WEST-Queen western blot detection kit, iNtRON Biotechnology), and blots were analyzed using a LAS 4000 mini (GE Healthcare Life Sciences). The intensity of each band was measured using optical densitometry of the analysis system (Multi Gauge version 3.0; Fujifilm, Tokyo, Japan).

### Statistical analysis

All data are expressed as mean ± standard error of the mean. Statistical significance of differences between groups was calculated using one-way and two-way analysis of variance followed by Tukey’s multiple comparisons test to determine the individual and interactive effects of FUS on immunochemistry and behavioral analysis. *P* < 0.05 was considered significant. All statistical analyses were performed using SPSS (Version 20, SPSS Inc., Chicago, IL, USA) and GraphPad Prism 5 software (GraphPad Software Inc., San Diego, CA, USA).

## Results

### Confirmation of cholinergic degeneration by SAP

**Fig. 2:**
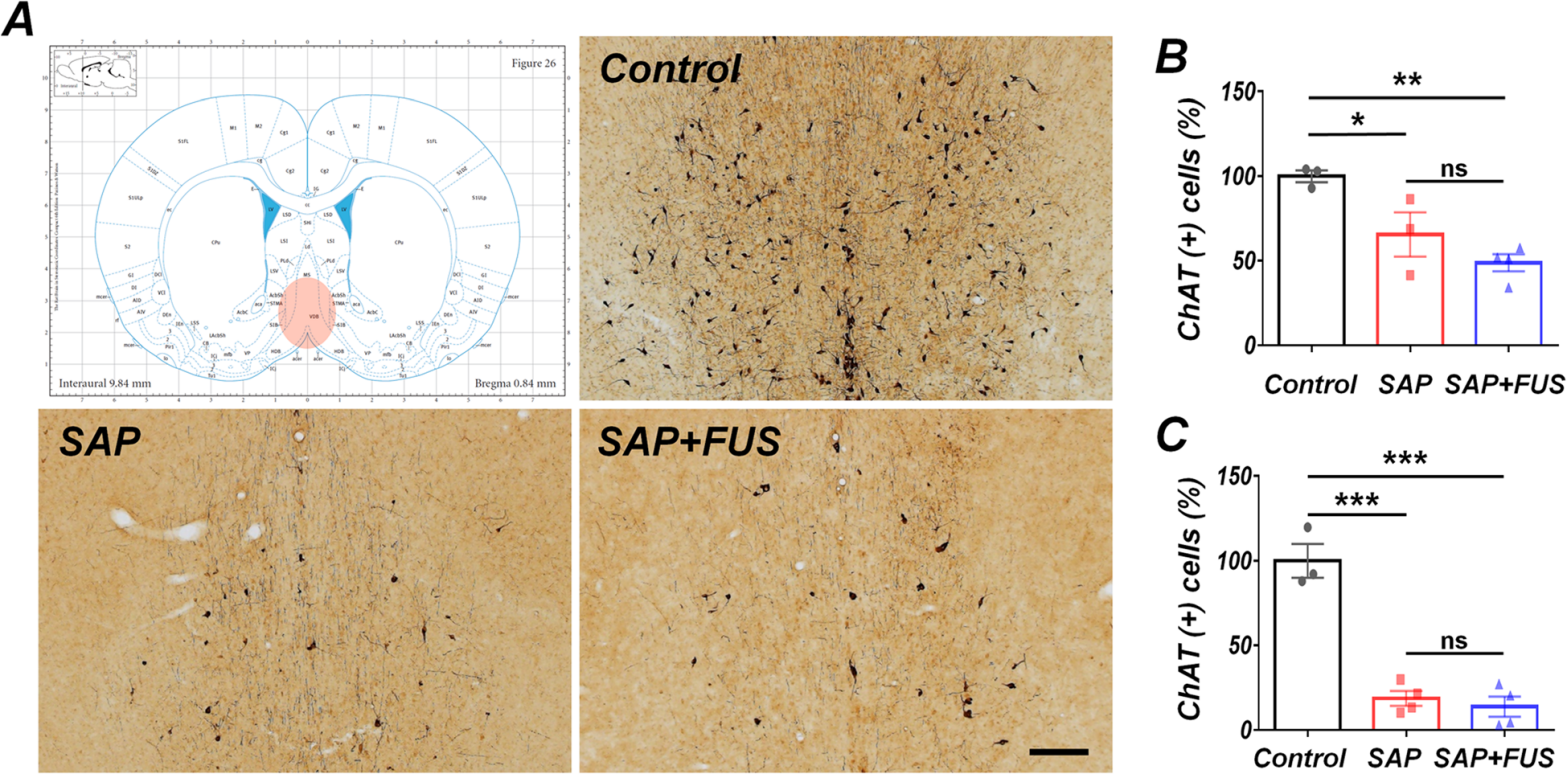
Dementia modeling confirmation of cholinergic lesion with 192 IgG-saporin reduces ChAT in MS/DB. **a** Representative histological sections showing the effect of the cholinergic lesion on MS/DB. The number of ChAT-positive cells was significantly decreased in both the SAP and SAP+FUS groups compared with that in the control group. Scale bar represents 200 μm. **b** Five days after sonication and **c** 18 days after sonication, bar graph represents ChAT-positive cells in MS/DB. Data are expressed as mean ± SE. *n* = 3–4 for each group. *P < 0.05, **P < 0.01, ***P < 0.001; one-way ANOVA with Tukey’s multiple comparisons test

To confirm cholinergic degeneration in our model, we quantified ChAT-immunopositive cells in MS/DB of each group of rats. Five days after sonication, compared with the control group (100 ± 3.5%), both the SAP (65.5 ± 13.1%; *P <* 0.05) and SAP+FUS (48.6 ± 5.05; *P <* 0.01) groups displayed a significantly reduced number of ChAT-immunopositive neurons (Fig. 2b). Eighteen days after sonication, compared with the control group (100 ± 10), both the SAP (18.7 ± 4.3; *P <* 0.001) and SAP+FUS (13.89 ± 5.9; *P <* 0.001) groups had significantly fewer ChAT-immunopositive neurons and less neuronal damage to cholinergic neuron bodies (Fig. 2c).

These results indicate that the number of cholinergic neurons were decreased in all groups at 5 and 18 days, which provide supporting evidence that dementia model using SAP was effective.

### FUS affects AChE activity in a dementia rat model

**Fig. 3:**
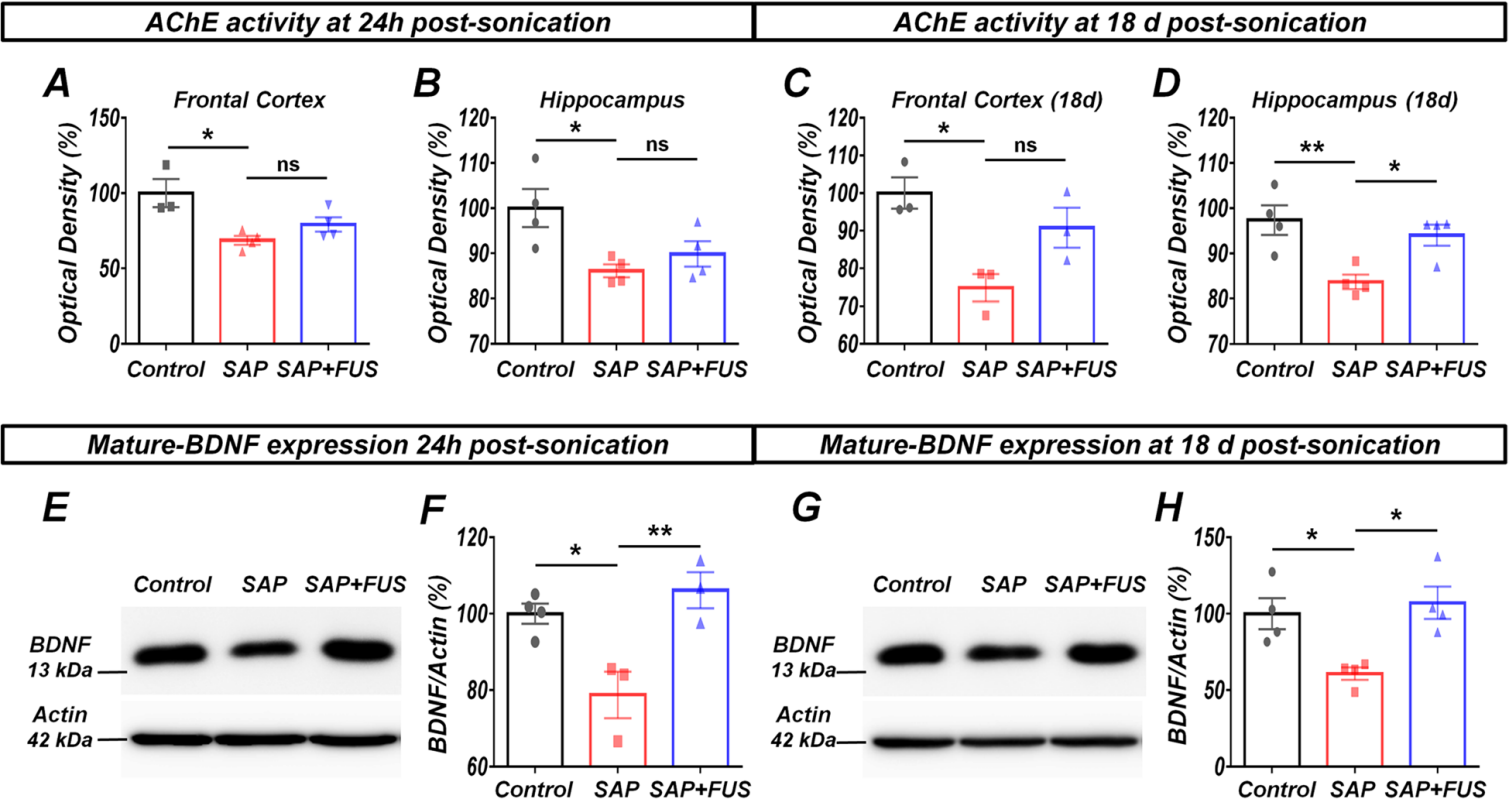
FUS increases AChE activity BDNF expression levels in a dementia rat model. **a** Twenty-four hours after sonication, AChE activity was significantly decreased in FC and **b** the hippocampus. **c** Eighteen days after sonication, FUS-mediated BBB opening induced a significant increase in AChE activity in FC and **d** the hippocampus. **e** Immunoblotting analysis shows BDNF protein expression levels in the hippocampus 24 h after sonication. BDNF levels in the FUS group increased significantly compared with those in the SAP and control groups. **f** Bar graph represents BDNF expression levels in the hippocampus. **g** Eighteen days after sonication, BDNF expression in the hippocampus in the FUS group increased significantly compared with that in the SAP and control groups. **h** Bar graph represents BDNF expression levels in the hippocampus. Data are expressed as mean ± SE. *n* = 3–4 for each group. *P < 0.05, **P < 0.01; one-way ANOVA with Tukey’s multiple comparisons test

To determine whether FUS affects cholinergic neuronal activity, we quantified AChE activity in each group. Twenty-four hours after sonication, AChE activity was significantly reduced in the SAP group in the frontal cortex (FC; 68.61 ± 3.02%; *P <* 0.05) and hippocampus (86.12 ± 1.43%; *P <* 0.05) compared with that in the control group (Fig. 3a, b).

Eighteen days after sonication, AChE activity was significantly decreased in the SAP group in the FC (74.85 ± 3.62%; *P* < 0.05) and hippocampus (83.70 ± 1.61%; *P* < 0.01) compared with that in the control group (Fig. 3c, d). However, the AChE activity of the hippocampus was significantly increased in the SAP+FUS group (94.03 ± 2.33%; *P* < 0.01) compared with that in the SAP group. The AChE activity of the FC was increased in the SAP+FUS group (90.79 ± 5.30%; *P* = 0.09) compared with that in the SAP group, but there was no significant difference between the two groups (Fig. 3c, d).

As a result, cholinergic degeneration of MS induced decreased activities of AChE in both FC and hippocampus at 24 h and 18 days. The effect of FUS treatment in AChE activities was only observed in the hippocampus at 18 days.

### FUS increases mature-BDNF expression in a dementia rat model

BDNF acts on specific neurons by promoting neurogenesis, which is crucial for long-term memory. To examine the effects of FUS on BDNF expression in the hippocampus, we performed immunoblotting analyses using brain samples from the hippocampal region obtained at 24 h and 18 days after sonication. The BDNF gene produces immature BDNF protein (17~32 kDa) and BDNF mature form (~ 13 kDa) by intracellular and extracellular proteases (Additional file 1: Figure S2) [39]. At both time points, compared with the control group, the SAP group (24 h: 80.15 ± 6.16%; 18 days: 60.79 ± 4.09%; *P* < 0.05) exhibited a significantly reduced expression level of mature-BDNF in the hippocampus, whereas compared with the SAP group, the SAP+FUS group (24 h: 108 ± 4.81%; *P* < 0.01; 18 days: 73.37 ± 10.63%; *P* < 0.05) showed a significantly increased level of mature-BDNF (Fig. 3e–h).

The cholinergic degeneration of MS induced decreased expression level of BDNF in the hippocampus at 24 h and 18 days. In contrast, FUS could upregulate the BDNF at the same time points.

### FUS affects EGR1 activity in a dementia rat model

**Fig. 4:**
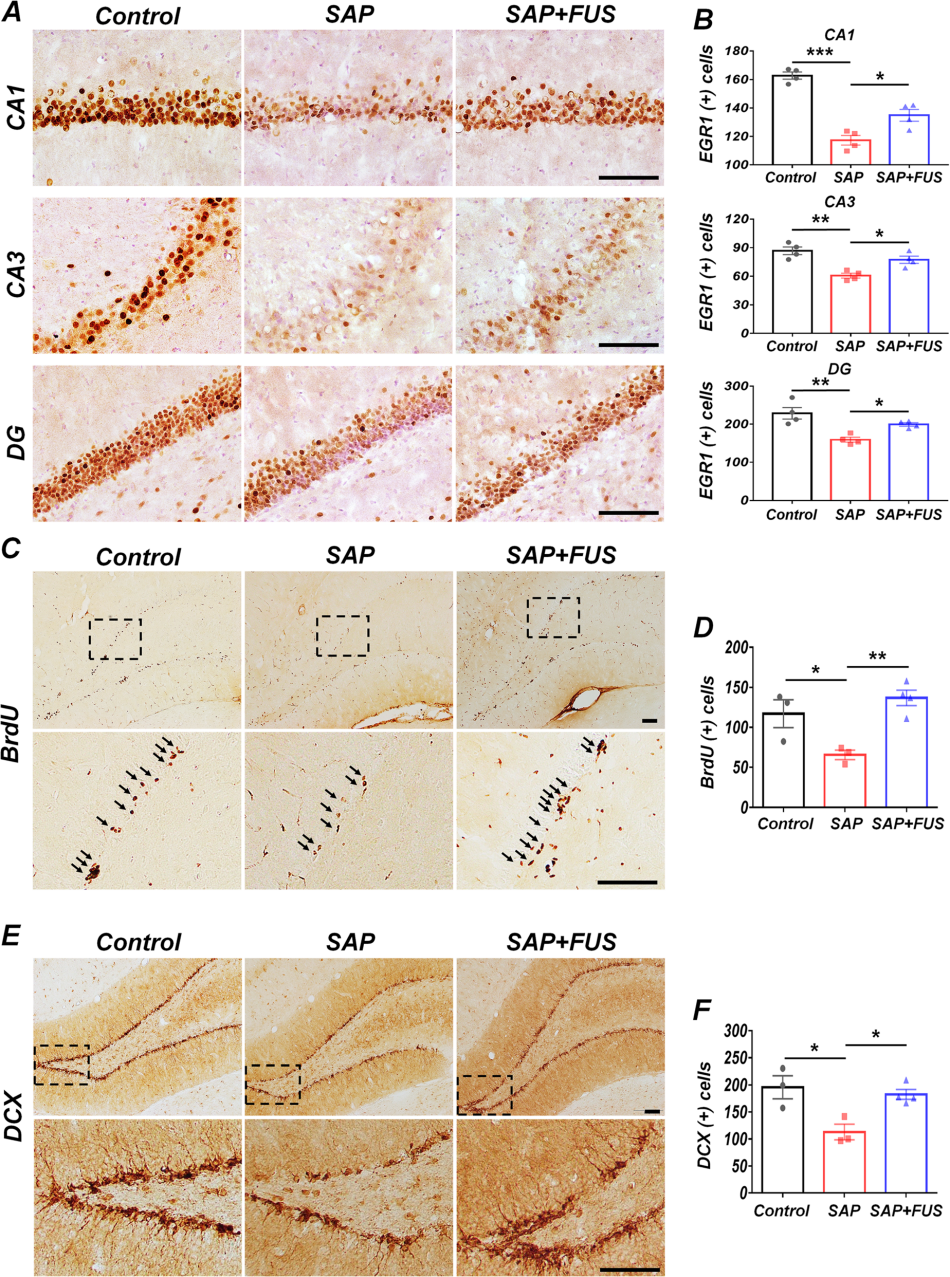
FUS upregulates EGR1 and proliferates neuroblast production in a dementia rat model. **a** Representative EGR1-stained brain sections 5 days after sonication. Compared with the SAP and control groups, the SAP+FUS group showed a significant increase in EGR1-positive cells in CA1, CA3, and DG of the hippocampus. Scale bar represents 100 μm. **b** Quantification of bar graph displays the number of EGR1-positive cells in CA1, CA3, and DG of the hippocampus. **c** Representative BrdU-stained brain sections 5 days after sonication. Compared with the SAP and control groups, the SAP+FUS group showed a significant increase in BrdU-positive cells in the SGZ of the DG of the hippocampus. Scale bar represents 100 μm. **d** Bar graph displays the number of BrdU-positive cells in SGZ of DG. **e** Representative DCX-stained brain sections 5 days after sonication. Compared with the SAP and control groups, the SAP+FUS group showed a significant increase in DCX-positive cells in SGZ of DG. Scale bar represents 100 μm. **f** Bar graph displays the number of DCX-positive cells in SGZ of DG. Data are expressed as mean ± SE. *n* = 3–4 for each group. *P < 0.05, **P < 0.01, ***P < 0.001; one-way ANOVA with Tukey’s multiple comparisons test. The scale bar represents 100 μm

EGR1, a transcriptional regulator, is extensively used as a marker for neuronal plasticity. To investigate whether FUS affects the transcription factor of EGR1 expression at 5 days after sonication, the number of EGR1-positive cells was visualized using immunohistochemistry. The SAP group exhibited a significantly lower number of EGR1-positive cells in CA1 (117 ± 4; *P <* 0.001), CA3 (67 ± 9; *P <* 0.01), and DG (159 ± 6; *P <* 0.01) of the hippocampus than the control group (CA1, 163 ± 2; CA3, 87 ± 4; DG, 229 ± 15). However, the EGR1 activity in the FUS group indicated a significant increase in CA1 (135 ± 4; *P* < 0.05), CA3 (77 ± 4; *P* < 0.05), and DG (199 ± 5; *P* < 0.05) compared with that in the SAP group (Fig. 4a, b).

These results indicate that cholinergic degeneration of MS caused the decreased activities of EGR1 in hippocampal region at 5 days, and FUS significantly upregulated the activities of EGR1 than SAP group.

### Effect of FUS exposure on the proliferation and neuroblast production in DG

The rats in each group were sacrificed 5 days after bilateral sonication of the hippocampal regions. Twenty-four hours after sonication, BrdU labeling was performed for 4 consecutive days for each group to observe progenitor cell proliferation in SGZ of DGs. We observed a decrease in the number of BrdU-positive cells in the SAP group (65 ± 6; *P* < 0.05) compared with that in the control group (117 ± 18), while this number was significantly increased in the SAP+FUS group (137 ± 10; *P* < 0.01) compared with that in the SAP group (Fig. 4c, d).

To investigate whether FUS affects newly generated immature neurons, the number of neuroblasts was visualized using doublecortin (DCX, a marker for neurogenesis) immunohistochemistry. Compared with the control (196 ± 21; *P* < 0.05) and SAP+FUS (183 ± 9; *P* < 0.05; Fig. 4e, f) groups, the SAP group exhibited a significantly reduced number of DCX-positive cells in DG of the hippocampus (113 ± 14).

It has been suggested that cholinergic degeneration of MS showed decreased activities of proliferation and neuroblast production in SGZ at 24 h and 18 days, which was reversed by FUS.

### FUS affects neurogenesis in a dementia rat model

**Fig. 5:**
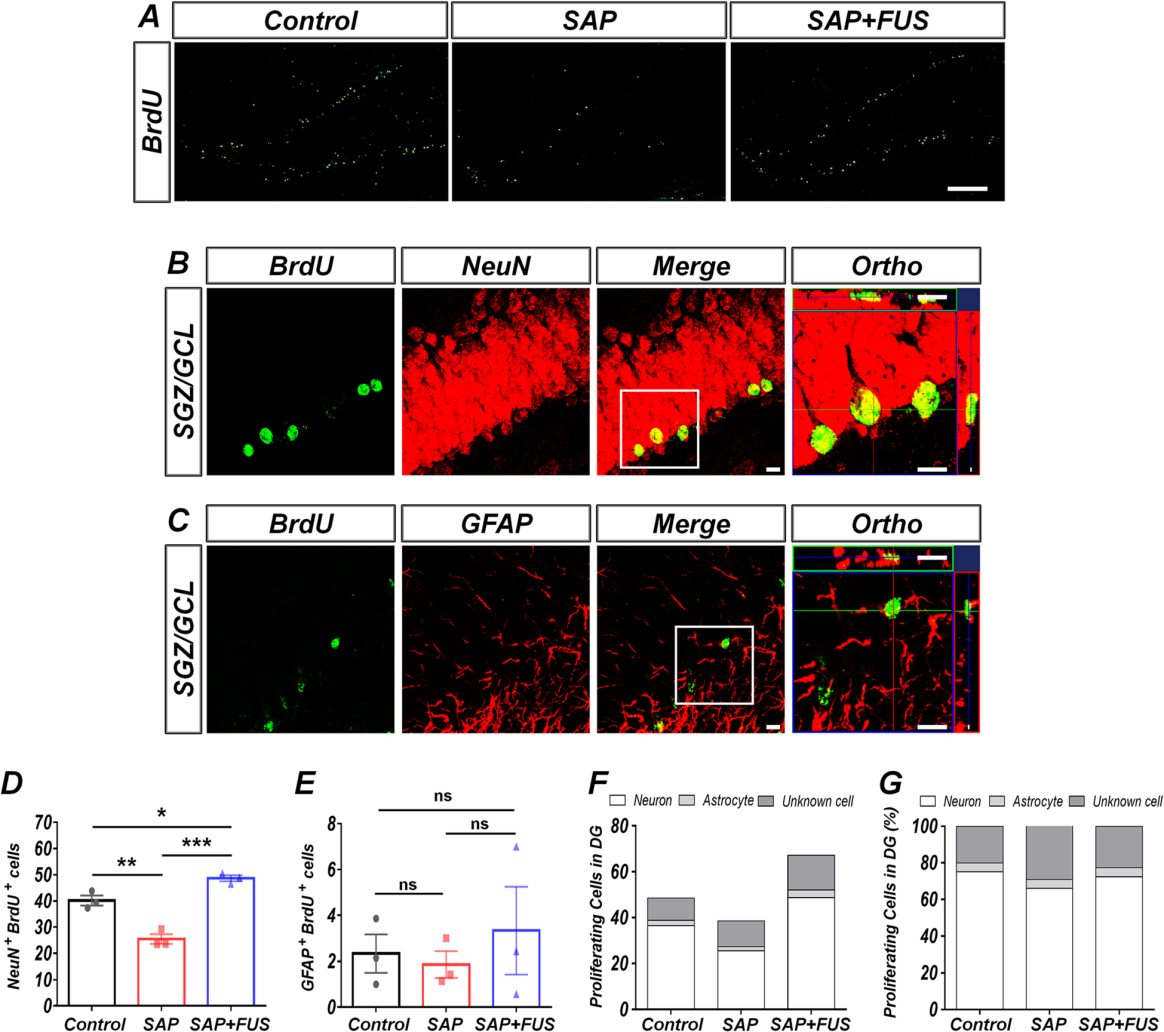
FUS increases neurogenesis and does not affect gliogenesis in a dementia rat model. **a** Representative photographs show the distribution of survived proliferation of BrdU-labeled positive cells in SGZ/GCL of DG of the hippocampus 18 days after sonication. The scale bar represents 200 μm. **b** Representative photographs of BrdU (green, a proliferative cell marker) and NeuN (red, a neuron marker) and **c** BrdU (green, a proliferative cell marker) and GFAP (red, an astrocyte marker) double-labeled cells in SGZ/GCL of DG of the hippocampus at 18 days after sonication. The scale bar represents 20 μm. **d** Quantification of BrdU and NeuN double-labeled cells. Compared with the SAP and control groups, the SAP+FUS group showed a significant increase in BrdU/NeuN-positive cells. **e** No significant differences were found in the numbers of BrdU/GFAP-positive cells among the groups. **f** Survived newly generating cells were determined, indicative of neurogenesis and gliogenesis. **g** The overall proportion of cells with the SGZ/GCL phenotype in DG of the hippocampus among the groups. Data are expressed as mean ± SE. *n* = 3–4 for each group. *P < 0.05, **P < 0.01; one-way ANOVA with Tukey’s multiple comparisons test

To determine the phenotypic characterization of BrdU-positive cells 18 days after sonication, sections were analyzed 2 weeks after the last injection of BrdU: neuronal phenotypes were identified by double-immunofluorescence labeling for NeuN and BrdU and glial phenotypes by double-immunofluorescence labeling for GFAP (astrocyte-specific marker) and BrdU (Fig. 5a–c). Compared with the control group (40 ± 2), the SAP group (25 ± 2; *P* < 0.01) displayed significantly reduced neurogenesis (NeuN+/BrdU+) in SGZ/GCL of DG. Compared with the SAP group, the SAP+FUS group (49 ± 1; *P* < 0.001) featured a significantly increased number of co-expression cells (NeuN+/BrdU+) in DG. No significant differences in gliogenesis (GFAP+/BrdU+) (Fig. 5e) and the phenotypes of BrdU-positive cells expressing NeuN or GFAP in DG (Fig. 5g) were identified among the groups.

The decreased AHN induced by cholinergic degeneration of MS was enhanced by FUS at 18 days. Interestingly, gliogenesis was not affected by FUS.

### FUS improved performance in the MWM task

**Fig. 6:**
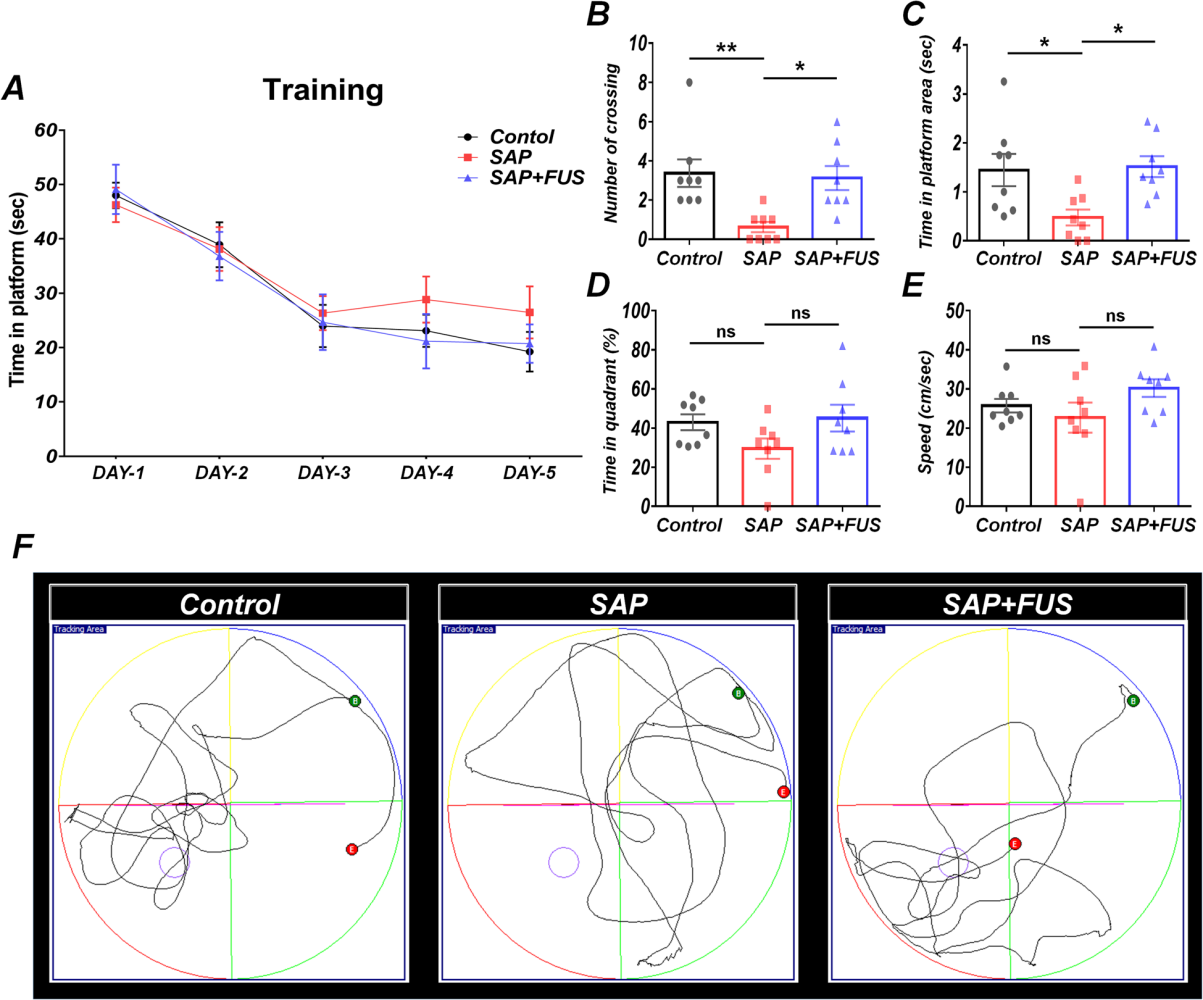
FUS improves cognitive function and spatial memory*.* **a** Learning curves of 5 consecutive days. Latency indicates the time required for the rat to find the escape platform during training trials. During the 5 days of training, the time to find the platform for rats in the control, SAP, and SAP+FUS groups gradually decreased. **b** In the probe test, rats in the SAP+FUS group crossed the platform area a significantly higher number of times (**c**) and spent a significantly longer time in the platform zone than did rats in the SAP or control groups. **d** Though rats in the SAP+FUS group had a longer time spent in the target quadrant zone, there were no significant differences among the groups. **e** No significant differences were found in the swim speed among the groups. **f** Trajectories of rats in the Morris water maze tests in the control group, SAP group, and SAP+FUS group. Data are expressed as mean ± SE. *n* = 8 for each group. *P < 0.05, **P < 0.01; one-way and two-way ANOVA with Tukey’s multiple comparisons test

To investigate the effects of FUS on memory and cognitive function, rats (*n* = 8/group) underwent training for MWM for 5 consecutive days (14 days post-modeling). All groups showed a gradual decrease in escape latency to the platform from the first day to the last day of the training (Fig. 6a).

In the MWM probe test, rats in the SAP group made significantly a lower number of crossing over (0.62 ± 0.26; *P* < 0.01) and spent less time in the platform area (0.47 ± 0.16; *P* < 0.05) compared with those in the control group (crossing over, 3.37 ± 0.7; platform area, 1.44 ± 0.32). In contrast, rats in the SAP+FUS group showed a significantly higher number of crossing over (3.12 ± 0.61; *P* < 0.05) and time in the platform area (1.51 ± 0.21; *P* < 0.05) compared with those in the control group (Fig. 6b, c). However, the time spent in the target quadrant and movement speed were not significantly different among the groups (Fig. 6d, e).

These results implicate that cholinergic degradation in MS reduces the memory and cognitive function, but FUS can reverse the impairment.

## Discussion

Although FUS may remedy the impermeability of BBB to pharmacotherapy, the effects of FUS on cholinergic function and AHN have not been elucidated in cholinergic-deficient conditions. Cholinergic systems regulate memory processing and cognitive function and link the memory circuit constituted by FC, hippocampus, and MS [40, 41]. The present study was designed to measure and analyze the potential effects of FUS in a rat model of dementia mimicking the BFC depletion in AD. The cholinergic dysfunction in this model was induced by intraventricular injection of SAP. This immunotoxin acts by coupling the ribosome-inactivating protein to a monoclonal antibody, which has a low affinity for the nerve growth factor receptor p75, located on BFC neuron bodies [42, 43]. In this study, we examined the cholinergic degeneration in this model, and our results revealed that the number of ChAT neurons was significantly reduced in MS of the SAP and SAP+FUS groups both 5 days and 18 days after FUS (Fig. 2), which proves that cholinergic dysfunction was well-established in this rat model, and the results are consistent with our previous results [33–36, 44, 45].

Because the reduction in AChE activity by cholinergic lesions in the hippocampus is closely correlated with Ach and AChE following compensatory mechanisms induced by SAP, we observed AChE activity in FC and the hippocampus [46] to examine the effects of FUS. Interestingly, the FUS group also exhibited decreases in the activity at 24 h, suggesting that sonication did not acutely affect AChE activity at 24 h (Fig. 3a, b). However, the SAP+FUS group showed a significantly increased AChE activity in the hippocampus 18 days after sonication (Fig. 3c, d), which implies that the FUS-mediated BBB opening resulted in the recovery of AChE levels.

EGR1 can activate AChE gene expression by binding to the AChE promoter. Overexpression of acetylcholine and AChE is critical for upregulating proliferative activity and subsequent neurogenesis [46]. Furthermore, acetylcholine modulates hippocampal long-term potentiation (LTP), thereby stimulating cholinergic neurons and enhancing hippocampal LTP [47, 48]. Another factor contributing to neurogenesis is BDNF, which has gained attention for its role in the regulation of synaptic transmission and plasticity, and neural circuit function in the CNS [49]. An insufficient supply of endogenous BDNF leads to neurodegeneration, cognitive impairment, and sharp decreases in neuronal proliferation in SGZ [50, 51]. Furthermore, dysfunction in the cholinergic forebrain system diminishes AHN in DG [52]. FUS increases neurogenesis in wild-type mice [29], and the effect of BDNF endures even 24 h after FUS treatment [24]. Because the correlation between BDNF and AHN has been already proven in previous research [53], our goal was to observe changes in BDNF and EGR1 activities in this model and how those factors could recover via FUS.

Consistent with previous observations, we demonstrated that the cholinergic-deficient conditions in the SAP group significantly reduced BDNF expression levels in the hippocampus (Fig. 3e, f) [54, 55]. The present study further demonstrated that the FUS-mediated BBB opening elevated BDNF expression both 24 h and 18 days after sonication of the hippocampus (Fig. 3f–h) and improved neurogenesis in GCL/SGZ of DG (Fig. 5d). These results indicate that FUS can promote BDNF expression 24 h after sonication, thereby confirming the results of previous research [24]. The mature BDNF primarily binds to the TrkB receptor, which plays a role in the development, maintenance, and differentiation of neurons and cell survival [56–58]. The maintenance of elevated expression of BDNF 18 days after sonication suggests that BDNF continuously regulates neurogenesis, synaptic plasticity, and membrane excitability [59, 60]. Our results indicate that diminished cholinergic input to the hippocampus reduces BDNF expression; FUS-mediated opening of BBB reverses these effects by stimulating hippocampal BDNF expression, which consequently regulates AHN positively in cholinergic degeneration [4].

Compared with the control group, the SAP group displayed decreased levels of EGR1 in the hippocampus (Fig. 4a), whereas EGR1 activity in the FUS group was significantly increased compared with that in the SAP group. Prior evidence has demonstrated that extracellular BDNF activates ERK expression by TrkB neurotrophin receptor [61]. The activation of transcription factors, such as CREB and IEGs including *c-fos* and *Egr1*, is followed by increased ERK phosphorylation [62]. This activation may play a critical role in BDNF upregulation induced by FUS, which could potentially contribute to the upregulation of EGR1 (Fig. 4a). In many studies, EGR1 transcription factors have been demonstrated to be major regulators and mediators of synaptic activity and plasticity under certain physiological conditions [63, 64]. Thus, our findings support prior evidence that BDNF facilitates the return of EGR1 to normal levels.

Our data support the theory that forebrain acetylcholine affects AHN, and a selective cholinergic lesion of the BFC system induces a decrease in BrdU, EGR1, DCX, and AChE levels; therefore, these findings indicate a reduction in proliferation and neuroblast production in SGZ and a decrease in hippocampal acetylcholine activity, respectively [35]. We found that the FUS-mediated BBB opening led to an increase in BDNF, EGR1, and AHN levels, which lead to an improvement in cognitive function.

Based on results from the behavioral test, we could also confirm that FUS enhanced memory and cognitive function. The performance of all rats in all groups gradually improved across 5 days of MWM training, suggesting that rats with cholinergic dysfunction have a similar level of learning capacity and escape latency compared with wild-type rats (Fig. 6) [34, 35]. In the probe test, when compared with the control and FUS groups, the SAP group displayed a diminished MWM performance 72 h after final training, as measured by the number of crossing over the platform area and time spent, which complements the findings of previous studies [33–36, 44]. However, FUS improved spatial memory, and cognition correlated with increases in EGR1, BDNF, and AHN. According to a recent study, increases in both AHN and BDNF levels affected memory improvement, similar to the effects of exercise in AD transgenic mouse [65]. However, increases in AHN alone did not have any effect [65]. Significant differences in speed were not observed among the groups, suggesting that there are no SAP-induced differences in motor function (Fig. 6e), which is consistent with our previous findings [35].

Although the data herein showed remarkable effects of FUS in this rat model, this study has several limitations that should be addressed in future research. We fixed sonication parameters to induce 0.25 MPa of acoustic pressure, which was adopted from our previous study [66]. However, recent studies have used an acoustic feedback system based on the passive cavitation detector to prevent tissue damage. This technique may guarantee appropriate sonication power and could be suitable for clinical application. Our previous study demonstrated that a FUS-mediated BBB opening could be safely and effectively performed within certain parameters [66]. Moreover, we used MRI to confirm BBB opening (T1W) without cell edema (T2W) after sonication (Fig. 1e, f). Furthermore, we observed increased BDNF expression only at 24 h and 18 days after sonication so different time points between 24 h and 18 days could be further studied to assess the changes in BDNF. In addition, we observed the recovery effects of FUS on EGR1 activities at 5 days in the model; thus, based on these results, we could assume that neuroblast production and cell migration might have maintained [53]. We could anticipate LTP and synaptic strength would recover.

## Conclusions

**Fig. 7:**
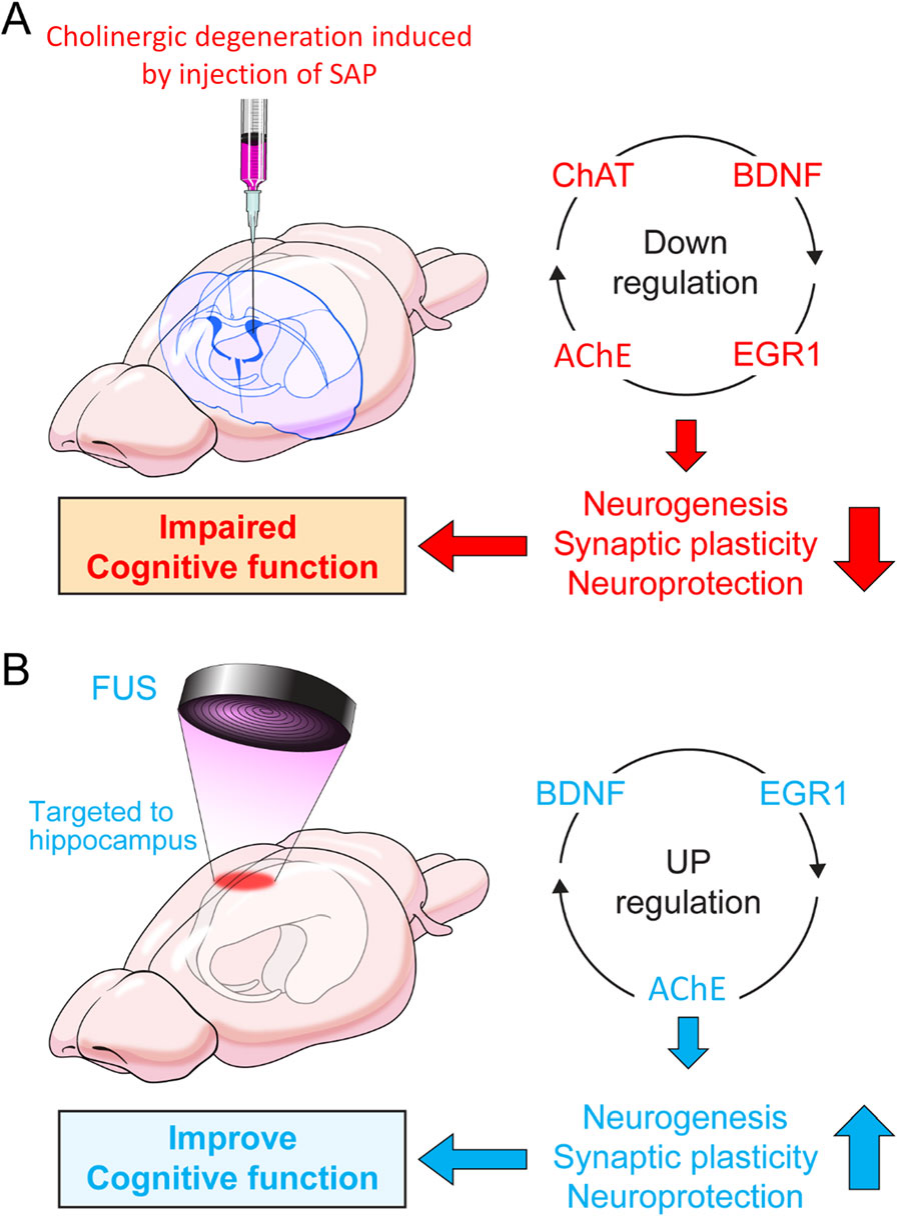
Diagrammatic summary of the relationship between cholinergic degeneration and FUS treatment*.* **a** Cholinergic degeneration induced by injecting 192 IgG-saporin (SAP) into the lateral ventricle downregulated the expression level of ChAT, BDNF, EGR1, and AChE, thereby causing impaired cognitive function by decreases of neurogenesis, synaptic plasticity, and neuroprotective function. In contrast, **b** FUS treatment targeting bilateral hippocampus improved cognitive function by upregulation of BDNF, EGR1, and AChE

In the present study, we demonstrated that animals with BFC hypofunction causing spatial memory impairment exhibit a reduction in cholinergic activity, neurogenesis, and BDNF and ERG1 expression levels (Fig. 7a). In contrast, FUS treatment increased AHN and improved spatial memory in cholinergic degeneration conditions. This improvement may be mediated by the upregulation of BDNF, EGR1, and AChE levels in the hippocampus, which is a critical factor for regulating AHN, synaptic plasticity, and neuroprotection (Fig. 7b). Because patients with AD have impaired cholinergic neurons and AHN starting at the early stages, FUS treatment may restore AHN and have a protective effect against neurodegeneration. Moreover, as FUS has been shown to be effective in increasing AHN, it could also contribute to increased permeability of BBB for drug delivery, and both these effects could be potential therapeutic strategies for AD.

## Supplementary information

**Additional file 1: Figure S1.** The schematic of the FUS experimental setup. **Figure S2.** Immunoblot of BDNF of multiple bands at mature-BDNF (~ 13 kDa) and immature-BDNF (17~32 kDa) were observed at (A) twenty-four hours after sonication and (B) eighteen days after sonication.

## Data Availability

The datasets used and/or analyzed during the current study are available from the corresponding author on reasonable request.

## Abbreviations

AHN: Adult hippocampal neurogenesis
AChE: Acetylcholinesterase
AD: Alzheimer’s disease
ANOVA: Analysis of variance
BBB: Blood-brain barrier
BDNF: Brain-derived neurotrophic factor
BFC: Basal forebrain cholinergic
ChAT: Choline acetyltransferase
CNS: Central nervous system
DCX: Doublecortin
DG: Dentate gyrus
FC: Frontal cortex
GCL: Granular cell layer
FUS: Focused ultrasound
MRI: Magnetic resonance imaging
MS/DB: Medial septum-diagonal band complex
MWM: Morris water maze
PBS: Phosphate-buffered saline
SAP: 192 IgG-saporin
SGZ: Subgranular zone
