# Supplementary material for: Focused ultrasound-induced blood-brain barrier opening improves adult hippocampal neurogenesis and cognitive function in a cholinergic degeneration dementia rat model
Source: Alzheimers Res Ther. 2019 Dec 27;11:110. doi: 10.1186/s13195-019-0569-x (PMC6933667; doi:10.1186/s13195-019-0569-x)
Supplement: Supplementary file 1 — Additional file 1: Figure S1. The schematic of the FUS experimental setup. Figure S2. Immunoblot of BDNF of multiple bands at mature-BDNF (~ 13 kDa) and immature-BDNF (17~32 kDa) were observed at (A) twenty-four hours after sonication and (B) eighteen days after sonication. [file 13195_2019_569_MOESM1_ESM.docx]

**Supplementary figures**


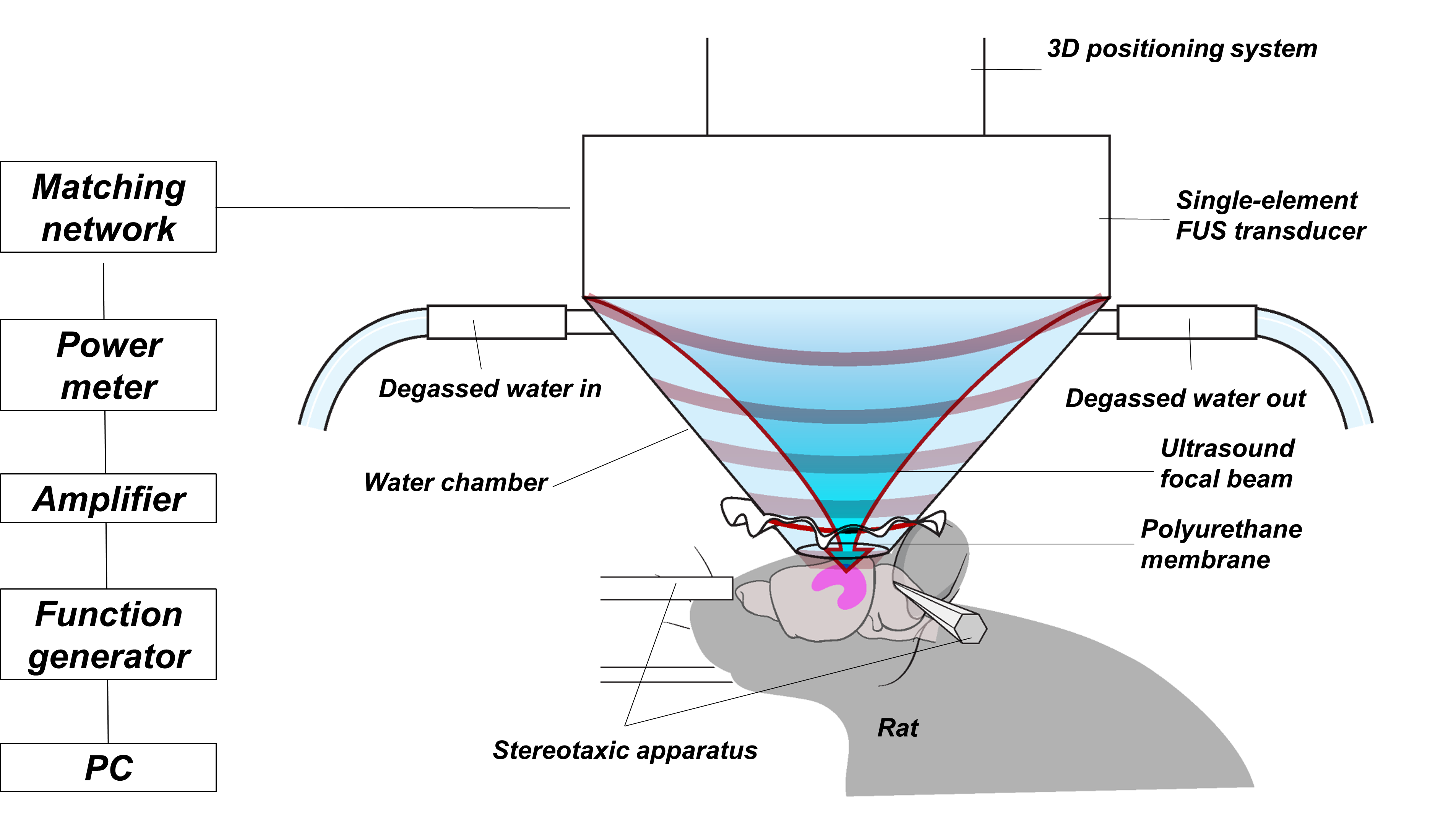


Fig. S1. The schematic of the FUS experimental setup.


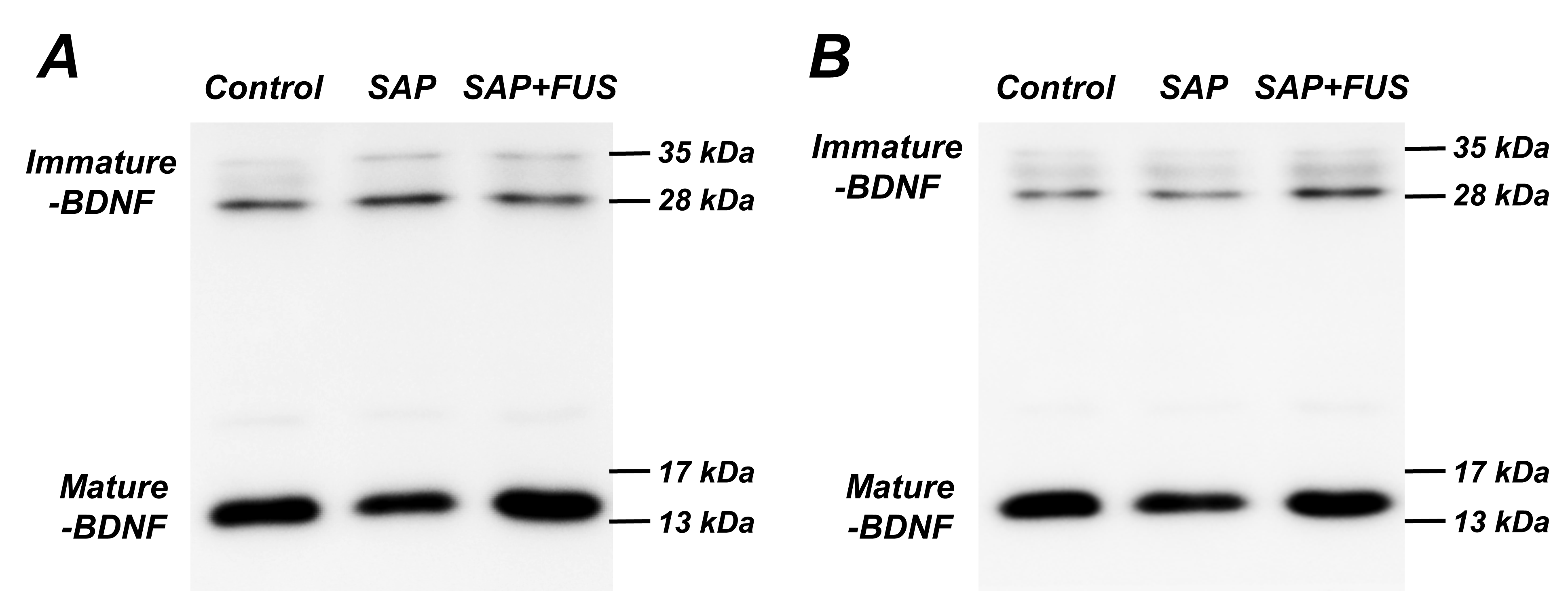


Fig. S2. Immunoblot of BDNF of multiple bands at mature-BDNF (~13 kDa) and immature-BDNF (17~32 kDa) were observed at (A) twenty-four hours after sonication and (B) eighteen days after sonication
